# A Pilot Study on Approach Bias Modification in Smoking Cessation: Activating Personalized Alternative Activities for Smoking in the Context of Increased Craving

**DOI:** 10.1007/s12529-021-10033-x

**Published:** 2021-10-25

**Authors:** Si Wen, Helle Larsen, Reinout W. Wiers

**Affiliations:** 1 Addiction Development and Psychopathology (ADAPT)-Lab, Department of Developmental Psychology, University of Amsterdam, Postbus 15916, 1001 NK Amsterdam, the Netherlands; 2 Research Priority Area Yield, University of Amsterdam, Amsterdam, the Netherlands; 3 Center for Urban Mental Health, University of Amsterdam, Amsterdam, the Netherlands

**Keywords:** Cognitive bias modification, Approach bias, Alternative activities, Craving, Smoking cessation

## Abstract

**Background:**

The act of smoking has been associated with the automatic activation of approach biases towards smoking-related stimuli. However, previous research has produced mixed findings when smokers are trained to avoid such smoking-related stimuli through the application of Approach Bias Modification (ApBM). As such, this study aimed to test an improved ApBM (ApBM +), where smokers were trained to approach personalized alternative activities for smoking in the context of increased craving, in addition to training smoking-avoidance responses.

**Methods:**

Sixty-seven daily smokers motivated to quit (*M* age = 29.27, 58.2% female) were randomly assigned to seven sessions of either ApBM + (*n* = 26), standard-ApBM (*n* = 19), or sham-ApBM (*n* = 22), after a brief motivational smoking intervention. Primary outcomes of approach biases for smoking and for alternative activities and secondary outcomes of smoking-related behaviors were assessed at pre-test, post-test, and 1-month follow-up.

**Results:**

Overall, no group differences by condition were demonstrated in changing approach biases or smoking-related behaviors at post-test and 1-month follow-up. A trend level indication for differences in changes of smoking-approach biases between sham-ApBM and ApBM + for relatively heavy smokers was found at post-test. This was primarily driven by a significant increase in smoking-approach biases within the sham-ApBM condition and a trend decrease in smoking-approach biases within the ApBM + condition.

**Conclusions:**

Our findings did not provide support for the current ApBM + concerning improved effects across the whole sample. Diverging training effects on approach biases for smoking in relatively heavy smokers warrants further research, for which we provide some suggestions.

**Supplementary Information:**

The online version contains supplementary material available at 10.1007/s12529-021-10033-x.

## Introduction

Why do so many smokers who are highly motivated to quit and aware of the negative health consequence related to smoking continually relapse? One possible explanation is that decision making in addictive behaviors can become biased by automatically activated cognitive-motivational processes (i.e., cognitive biases in the processing of substance-related cues [1, 2]). Smokers indeed have shown an approach bias for smoking-related cues, as indicated by faster action tendencies in approaching as opposed to avoiding smoking-related cues [3]. Further, this approach bias has been associated with craving, nicotine dependence, and relapse [3–5]. Taken together, an approach bias for smoking-related cues is thought to play an essential role in the maintenance of smoking behaviors and therefore constitutes an important target for smoking cessation interventions.

One form of Cognitive Bias Modification (CBM) is Approach Bias Modification (ApBM), an intervention developed to directly modify approach biases (see Wiers et al. [2] for a review). In the addiction context, a standard ApBM procedure trains participants to consistently avoid substance-related stimuli (e.g., someone smoking) and approach visually matched control stimuli (e.g., someone holding a visually matched object, like a pen). The assumed working mechanism of ApBM relies on the repeated pairing of substance cues with an action tendency to avoid, thereby overwriting substance-approach associations. In doing so, ApBM is suggested to facilitate a change in addictive behaviors, assuming individual motivation to change [6, 7]. To date, while the application of ApBM as an add-on in the treatment of alcohol use disorders (AUDs) has been largely successful [8–11], evidence for the effectiveness of ApBM in smoking cessation remains mixed. Specifically, two studies have produced relatively positive results: ApBM aided in the reduction of smoking behaviors [12, 13]; yet conversely, four other studies were unable to support ApBM as an effective tool in fostering smoking cessation, neither when delivered as a stand-alone intervention [14, 15] or when employed as an addition to traditional evidence-based smoking interventions [16, 17]. Moreover, no consistent evidence was found for a change in smoking-approach biases. Hence, there is room for improvement regarding ApBM as an (add-on) smoking intervention. The present study introduces two potential changes to help improve the effects of ApBM.

First, activating meaningful alternative responses, in addition to regular training of avoidance responses to smoking, may improve the effects of ApBM. One notable difference between the standard ApBM for treating AUD (alcohol-ApBM) vs. smoking cessation promotion (smoking-ApBM) relates to the employed control stimuli. For those abstaining from alcohol, the control stimuli in alcohol-ApBM is typically a non-alcoholic drink, representing a meaningful and what could be regarded as a generally relevant alternative behavioral choice. As a result, in alcohol-ApBM, not only a response of avoiding alcohol is trained but also a response of approaching meaningful alternatives [11]. However, the same cannot be said in smoking-ApBM; here, the control stimuli are routinely a visually matched control-object (e.g., a pen), meaning therapeutically, smoking-ApBM only trains an avoidance response to smoking, without training an appropriate approach response. Thus, neglecting to provide a relevant alternative behavioral choice in smoking-ApBM may be the contributing factor impeding the intervention’s efficacy compared to alcohol-ApBM.

Such rationale is supported by prior literature, stating that in order to break old habitual response, it is equally important to build new adaptive habits simultaneously [18]. Indeed, increased engagement in healthy alternative activities (e.g., physical activity) has been associated with a greater probability of successful cessation [19]. In line with this idea, two recent studies have integrated meaningful alternative stimuli within their smoking-ApBM as a category of stimuli to be approached [4, 18]. Specifically, a first study examined the effect of ApBM in adult smokers who were motivated to quit, using positive social interactions as a meaningful alternative approach-stimuli [4]. Application of this modified ApBM led to greater reductions in the smoking-approach biases over and above the sham training, and further, the reduction in smoking-approach biases significantly related to a longer duration of abstinence. In the second study, smokers presenting with depression who reported relying on smoking as a coping mechanism were trained to deal with their negative emotions in ways other than smoking through a variety of ApBM. For example, training participants to approach personalized alternative activities to deal with negative affect (e.g., cycling) [18]. The study findings showed that this modified ApBM led to a weaker association between negative emotion and smoking, a stronger decrease in depressive symptoms, and higher abstinence rates (at trend level) compared with the sham training. While both of the modified ApBM varieties showed promise, thus far, no direct comparison has been made between the modified versions and the standard ApBM.

A second alternation that may improve the effects of ApBM includes delivering ApBM in a smoking-related “hot” context. Two recent studies examining the role of attention within social anxiety indicated that contextually activating fear before receiving CBM improves the effectiveness of treatment in reducing anxiety symptoms [20, 21]. Translating these findings to the field of smoking cessation, it seems reasonable to suggest that training after exposure to real-life smoking cues, thereby triggering the craving responses may improve smoking-CBM efficacy. Some studies have indicated that substance-related cognitive biases are more pronounced under higher levels of craving [22, 23]. These results suggest that substance-related cognitive biases may play a more prominent role in craving-provoking environments (i.e., a context where the substance is most usually consumed). In other words, smokers may be particularly hampered by their smoking-approach biases in situations and environments that provoke higher levels of craving. Hence, learning to avoid smoking cues appears to be most relevant in a state of craving. Furthermore, according to the specific encoding theory [24], a newly learned behavior could be strengthened when learned in a context similar to where it is enacted or executed in real life. Thus, performing ApBM while being in a state of increased craving might help the transfer ApBM training effects to daily life.

In sum, we identified two potential changes that may help to improve smoking-ApBM effects: (1) helping smokers to build new adaptive behavioral responses (e.g., to approach personally relevant alternative activities instead of smoking) and (2) training smokers in a smoking-related “hot” context (e.g., in a state of increased craving after exposure to personally relevant risk situations for smoking). In order to maximize the potential effect, we chose to combine both alterations aforementioned into a new variety of ApBM (ApBM +). Specifically, in ApBM + , prior to training, participants are first instructed to imagine themselves within a self-identified risk situation for smoking (i.e., setting the relevant context), intended to increase subjective craving. Once the context is established, participants are trained to avoid smoking pictures and approach self-selected alternative-activity pictures. We postulated that this procedure should both reduce smoking-approach associations and increase alternative activities-approach associations. As such, ApBM + would increase the likelihood of smokers engaging with the alternative activity for smoking when craving-related risk situations of smoking are experienced. Additionally, engagement in the alternative activities should automatically distract from smoking and, therefore, facilitate abstinence.

In this pilot study, we performed an initial test of ApBM + in adult smokers who were motivated to quit. Our first aim was to investigate whether ApBM + would change the two targeted mechanisms of smoking: reduce approach biases for smoking and increase approach biases for alternative activities. Additionally, our second aim was to explore the potential effects of ApBM + in changing smoking behaviors as add-on to a brief motivational smoking intervention. To test the training effects of ApBM + , we compared our new variant against standard-ApBM and sham-ApBM. Specifically, in the standard-ApBM, participants were trained to avoid smoking pictures and to approach neutral activity pictures within a neutral context; in the sham-ApBM, participants were trained to equally avoid and approach each of the picture categories again within a neutral context. We hypothesized that, when compared to standard-ApBM and sham-ApBM, ApBM + would result in (1) a weaker approach bias for smoking, (2) a stronger approach bias for alternative activities, and (3) better smoking-related behavioral outcomes (i.e., lower craving levels, lower breath carbon monoxide levels, less daily cigarette consumption, and higher 7-day point prevalence abstinence rates) at both post-test and 1-month follow-up. Moreover, our third aim was to explore whether severity of smoking would moderate the effects of ApBM + on all outcomes. Evidence suggests that heavy smokers showed stronger smoking-approach biases than light-to-moderate smokers [5, 25] and responded better to CBM [5]. Therefore, we expected stronger effects of ApBM + in relatively heavy smokers (defined as smoking more cigarettes per day).

## Method

### Study Design

This pilot represents a randomized parallel-group study, employing a 3 (Condition: sham-ApBM, standard-ApBM, and ApBM +) × 3 (Time: pre-test, post-test, and 1-month follow-up) mixed experimental design. Participants were randomly allocated to one of the three experimental ApBM conditions to receive seven training sessions. Prior to ApBM training, participants underwent a brief motivational smoking intervention that consisted of three sessions utilizing a combination of Cognitive Behavior Therapy and Motivational Interviewing [16]. Within the first session, participants formed implementation intentions to help identify personally relevant risk situations for smoking and alternative activities for smoking [26]. Once identified, these implementation intention outcomes were used to build personalized ApBM training and assessment protocols. The remaining two sessions were used to increase participants’ motivation to change prior to the start of the ApBM training, as evidence suggests that CBM works best for participants who are motivated to change [6]. Training outcomes of approach biases and smoking-related behaviors were repeatedly assessed at pre-test, post-test, and 1-month follow-up. This study was approved by the Ethics Review Board of the Faculty of Social and Behavioral Science of the University of Amsterdam (reference number: 2017-DP-8067).

### Participants

Adult smokers were recruited through online advertisements via our lab website (https://www.impliciet.eu/) and a community-based recruitment platform (https://www.proefpersonen.nl/), and through flyers, posters, and by word of mouth around the University of Amsterdam. The inclusion criteria stipulated that participants (1) were 18–65 years old, (2) reported smoking at least 5 cigarettes per day in the past half-year, (3) intended to quit smoking but had not yet quit, (4) had regular computer and internet access, (5) could read and speak Dutch, and (6) reported that they were not color blind. After online screening, 88 eligible participants attended a baseline visit at the lab, during which 11 participants were excluded because they were identified as non-smokers based on their breath carbon monoxide (CO) test (i.e., CO levels <  = 3 parts per million (ppm)). The cut-off score of 3 ppm of CO levels was adopted as previous studies have shown this score to most accurately distinguish non-smokers from smokers [27, 28]. Of the remaining 77 participants randomized to the intervention conditions, 10 dropped out at the beginning of training. As a result, 67 participants received the allocated training and completed the post-test (*n* = 26, 19, and 22 in the ApBM + , standard-ApBM, and sham-ApBM condition, respectively), and 56 participants completed the 1-month follow-up (*n* = 18, 17, and 21 in the ApBM + , standard-ApBM, and sham-ApBM condition, respectively). A participant flowchart can be found in Fig. S1 in Electronic Supplementary Material 1.

### Measures

The following demographic and smoking history information was collected: age, gender, the highest completed education level, duration of years of smoking, daily cigarette consumption in the past half-year, nicotine dependence, the number of previous quit-attempts, and readiness to change. Nicotine dependence was measured with the six-item Fagerström Test for Nicotine Dependence (FTND [29]). Sum scores ranged from 0 to 10, with a higher score reflecting more elevated levels of nicotine dependence. Internal consistency (Cronbach’s α) of FTND was 0.69 in this study. Readiness to change was measured using the 12-item Readiness to Change Questionnaire (RCQ [30]). Sum scores ranged from − 24 to 24, with a higher score reflecting an increased readiness to change. The internal consistency of RCQ was 0.68 in this study.

#### Primary Outcomes: Approach Biases

Approach biases (ApB) were measured with an online version of the Approach-Avoidance Task (AAT [16, 31, 32]). Task parameters (picture onset, response time window, inter-trial interval, etc.) followed the Boffo et al. protocol [31]. Within the task, three categories of pictures were used: 24 smoking pictures (e.g., someone smoking), 24 neutral-activity pictures (e.g., someone pressing keys), and 24 personalized alternative-activity pictures (e.g., someone meditating). The former two categories of pictures were taken from a previous study [33], and the latter category was provided by the participants (in the first session where forming personalized implementation intentions). In each trial, a picture was presented in the middle of the computer screen, with a three-degree tilt to the right or left. Participants were required to respond to (“pull” and “push”) the format of the picture (e.g., pull when tilted left) as fast as possible by pressing the corresponding keys on the keyboard (“U” and “N”). The push and pull responses were accompanied by a zooming effect: pulled pictures enlarged in size, pushed pictures shrunk, generating the sense of approach and avoidance, respectively [32]. The contingency between the picture format and the response was counterbalanced across participants. To assess ApB, the three categories of pictures were pushed and pulled equally often, with each picture presented four times, resulting in 288 total trials. The AAT started with 10 practice trials utilizing a grey square instead of the assessment stimuli. An ApB for each picture category was computed by subtracting the median response time to pull trials from that to push trials. Positive and negative scores represent an approach bias and avoidance bias, respectively.

Before computing ApB indices, the subsequent trial data were removed following Machulska et al. [13]: (1) incorrect trials (AAT at pre-test: 4.65%, AAT at post-test: 5.78%, AAT at 1-month follow-up: 4.39%) and (2) correct repetitions of incorrect trials. Bootstrapped split-half reliability estimates for each picture category of the AAT were obtained by using the *splithalf* package in R (version 0.5.2 [34]) with 5000 random splits (more details can be found in Electronic Supplementary Material 2). Reliability of the AAT was *r* = 0.25, *95% CI* = [0.09, 0.41] for smoking pictures; *r* = 0.23, *95% CI* = [0.06, 0.39] for alternative-activity pictures; and *r* = 0.23, *95% CI* = [0.05, 0.39] for neutral-activity pictures.

#### Secondary Outcomes: Smoking-Related Behaviors

Craving was measured by the 10-item Questionnaire on Smoking Urges (QSU-Brief [35]). Sum scores ranged from 10 to 70, with a higher score reflecting higher levels of craving. The internal consistency of QSU was 0.79 in this study. Daily cigarette consumption (DCC) was measured using the Timeline Follow Back method (TLFB [36]). The internal consistency of TLFB was 0.95 in this study. CO levels were measured with a smokelyzer (ToxCOTM, Bedfont Scientific Ltd, Kent, UK). Seven-day point prevalence abstinence (7D-PPA) was defined as not smoking at all in the past 7 days before the test (i.e., 0 on TLFB verified with CO levels <  = 3 ppm [27, 28]).

### Intervention

#### Brief Motivational Smoking Intervention

The brief motivational smoking intervention consisted of one session where participants formed implementation intentions and two Motivational Interviewing (MI) sessions. Each session lasted approx. 30 min. Eight trained research assistants (RAs) performed the interventions under the supervision of the second author (a cognitive behavioral therapist). All of the RAs had a master’s level education and had received MI training during their studies.

##### Formation of Implementation Intentions

This session was used to help participants in selecting personalized materials in four steps. First, participants were required to select their top four risk situations from a list of 13 common smoking risk situations (e.g., feel stressed [37]). Second, they were asked to write down three alternative activities for each chosen risk situation resulting in 12 alternative activities (e.g., meditating). Third, participants formed 12 implementation intentions in the form of if–then plans (e.g., if I feel stressed, then I will meditate [26]). Finally, participants chose pictures to represent their alternative activities (online search in the lab). Participants were required to provide two pictures for each alternative activity. Thus, in total, each participant provided 24 alternative activity pictures (see Electronic Supplementary Material 3 for details on criteria of picture selection). Table 1 shows an overview of the selected risk situations for smoking and the personalized alternative activities provided by the participants.

##### Motivational Interviewing (MI)

Two MI sessions (“preparation to quit” and “pre-quit”) were used to increase participants’ motivation to change, using an adapted version of a manual-guided protocol [16]. Motivational and cognitive-behavioral strategies were used to motivate participants to quit smoking through focusing on different aspects, such as benefits of quitting vs. risks of continued use and goal setting (e.g., set a quit date to affirm commitment). Participants’ risk situations for smoking and alternative activities generated in the session where forming their implementation intentions were also discussed.

#### Approach Bias Modification (ApBM)

There were seven ApBM sessions. Each session included a Mental Imagery Procedure (MIP), which lasted approx. 3 min, and an ApBM training, which lasted approx. 10 min.

##### Mental Imagery Procedure (MIP)

Each ApBM session started with a MIP. The real-MIP was only delivered in the ApBM + condition with the aim to increase participants’ craving before the training. The MIP was chosen for this study as MIP has been widely used in cue-reactivity research and demonstrated as an effective method to induce drug-related craving [38]. The MIP used the following procedure [39]. First, participants indicated current craving on a visual analog scale (VAS, 0–100, extreme craving). Next, the top four smoking risk situations selected previously by the participants appeared on the screen, and participants were instructed to choose one of which they felt most difficult to deal with at that moment. A pre-written script corresponding to the chosen risk situation, including explicit smoking urges, was then shown for 60 s, with the instruction to read the script carefully. Participants were then told to close their eyes and actively imagine the script they had just read for a further 30 s until they heard a stop signal. Lastly, participants were asked to write a short text to describe the situation they had just chosen, read, and imaged. They were then instructed to rate the vividness of their imagined image during the MIP on a VAS (0–100, extremely vivid) and indicate their current craving on an additional VAS. Participants in the standard-ApBM and sham-ApBM conditions received a sham-MIP. In this version, participants were provided with four pre-written neutral situations and scripts (e.g., rake leaves), with the instruction to choose one situation and imagine it as their training context. All the pre-written scripts were adapted from previous cue-reactivity research [39].

##### ApBM Training

Directly after the MIP, the ApBM training started. ApBM was adapted from the AAT [16, 31]. In the training session, 16 smoking pictures, 16 neutral-activity pictures, and 16 personalized alternative-activity pictures were used. Each training started with 10 practice trials showing a grey square picture followed by 192 training trials (each picture presented four times). The contingencies between the picture categories and responses were manipulated in the training conditions to train avoidance of smoking and approach of alternative activities or neutral activities. Specifically, in the ApBM + condition, 100% of the smoking pictures were presented in the push-format, 100% of the alternative-activity pictures in the pull-format, and neutral-activity pictures were presented equally often in the push and pull-format. In the standard-ApBM condition, 100% of the smoking pictures were presented in the push-format, 100% of the neutral-activity pictures in the pull-format, and alternative-activity pictures were presented equally often in the push and pull-format. In the sham-ApBM condition, all three categories of pictures were presented equally in the push- and pull-format (same as the AAT).

### Procedure

Those interested in participating were directed to the study website (https://www.lab.uva.nl/lotus/AAA_nl/page/C_home). Upon registration, participants read an information letter and submitted a digital consent form, through which they were fully informed as to the experimental design of the study noting the 33% chance to be assigned to a sham training condition. After submitting the consent form, participants were screened for eligibility. Eligible participants were scheduled to attend the baseline visit (Visit 1) at the lab. During which, participants’ CO levels were verified, indicating their smoking status, and they completed the pre-test (including demographics, smoking history, QSU, and TLFB). This was immediately followed by the session where the implementation intentions were formed. At Visit 2 (one day after Visit 1), participants first completed a baseline AAT and were randomized to one of the three intervention conditions (details on randomization procedure can be found in Electronic Supplementary Material 4). Afterwards, participants received the first MI session, at the end of which they selected a quit date in the following week. At Visit 3 (1 day before participants’ quit date; about 1 week after Visit 2), participants received the second MI session followed by the first ApBM training. Additionally, participants were required to do five ApBM training sessions at home. Each home session opened 24 h after the previous session was completed and stayed open for 24 h. Therefore, the finishing durations of the five home training sessions ranged from 5 to 15 days. At Visit 4 (1 day after the sixth training session was completed or expired), participants received the seventh ApBM training and completed the post-test (including AAT, CO, QSU, and TLFB). Finally, at Visit 5 (1-month follow-up), participants completed the follow-up test (including AAT, CO, QSU, and TLFB) and received a debriefing based on their allocated condition. Participants were compensated with € 40 or four course credits if they received the allocated intervention and post-test, and they were further compensated with an extra €10 or one course credit if they completed the follow-up test. A visualization of the study procedure can be found in Fig. S2 in Electronic Supplementary Material 5.

### Statistical Analyses

Based on previous studies, an effect of *f* = 0.250 was assumed for the sample size calculation of our primary outcomes (i.e., ApB) at post-test [8, 11]. Using G*Power [40], an *α* of 0.05 and a *β* of 0.80 suggested 63 participants would be needed to detect an interaction effect in the 3 (Condition) × 2 (Time: pre-test vs. post-test) analysis. Additionally, an effect of *f* = 0.175 and an attrition rate of 20% was assumed for the sample size calculation of our primary outcomes (i.e., ApB) at 1-month follow-up [12, 13], suggesting 166 participants should be included to detect an interaction effect in the 3 (Condition) × 2 (Time: pre-test vs. 1-month follow-up) analysis. However, due to slow recruitment and practical reasons, data collection ended when the sample size was sufficient to detect the expected training effect at post-test. Thus, we focused on the training effects on primary and secondary outcomes at post-test in this report. Regarding the training effects on all outcomes at 1-month follow-up, such results were only summarized within the study. All data analyses were performed by using SPSS 22.0 [41].

Given the exploratory nature of this study, training effects at both post-test and 1-month follow-up were primarily tested by using a complete case analysis method. Specifically, training effects at post-test were evaluated based on participants who completed outcomes at both pre-test and post-test (*N* = 67), and training effects at 1-month follow-up were evaluated based on participants who completed outcomes at both pre-test and 1-month follow-up (*N* = 56; see Fig. S1 for participant flowchart in Electronic Supplementary Material 1). To test the training effects on changes of our continuous primary and secondary outcomes (i.e., ApB, QSU, DCC, and CO) at post-test, a series of mixed-model ANOVAs were conducted with a 3 (Condition) × 2 (Time: pre-test vs. post-test) design. In case of large deviations of normality of the outcomes, non-parametric tests were also performed (i.e., related sample Wilcoxon signed-rank tests for Time effects and Kruskal–Wallis tests on difference scores for Condition effects). Since the non-parametric outcomes did not differ substantially (same conclusions) from the mixed-model ANOVAs, the latter were reported. Additionally, to test the training effects on our binary secondary outcome (i.e., 7D-PPA) at post-test, a Chi-square test was conducted. Furthermore, to explore whether severity of smoking (measured by using daily cigarette consumption in the past half-year) would moderate training effects at post-test, a series of linear regression analyses (for ApB, QSU, DCC, and CO) and a logistic regression (for 7D-PPA) were conducted. In each model, Condition, severity of smoking, and their interaction term were entered. Additionally, we entered the Z-standardized pre-test value of that outcome as a covariate when the outcome was continuous. Finally, all analyses mentioned above were also applied to test the training effects at 1-month follow-up.

Training effects at both post-test and 1-month follow-up were also tested using an intention-to-treat (ITT) analysis method as a sensitivity analysis. The ITT analyses were conducted based on the randomized sample (*N* = 77; see Fig. S1 for participant flowchart in Electronic Supplementary Material 1). Prior to the ITT analyses, missing data points were replaced using multiple imputations. Given that the results of the ITT analyses remained essentially the same in our primary complete case analyses, we only included the results of the complete case analyses in this report due to the pilot nature of this study and to keep the report concise. Details on ITT analyses and their results can be found in the Electronic Supplementary Material 6.

## Results

### Primary Analysis

The baseline characteristics and the training outcomes assessed at pre-test of the complete cases at post-test (*N* = 67) can be found in Tables 2 and 3. Comparison across the three training conditions revealed no significant baseline differences in relation to participants’ characteristics and training outcomes. To verify whether there were baseline ApB, one-sample *t*-test was conducted. Results showed that, overall, participants demonstrated neither an approach nor an avoidance bias for any of the picture categories (ApB for smoking: *M* = 12.01, *SD* = 56.51, *t*(66) = 1.74, *p* = 0.087; ApB for alternative activities: *M* = 0.12, *SD* = 54.51, *t*(66) = 0.02, *p* = 0.986; ApB for neutral activities: *M* = 2.75, *SD* = 52.77, *t*(66) = 0.43, *p* = 0.671).

**Table 1:** Overview of the selected risk situations for smoking and the personalized alternative activities provided by the participants (*N* = 67)

**Risk situations for smoking**	**Participants who selected the risk situations: *n* (%)**
When I am stressed	43 (64.18)
When I am at a party or in a cafe	43 (64.18)
When I am having a (work) break	39 (58.21)
After dinner	32 (47.76)
When I wake up in the morning	28 (41.79)
When I feel angry or frustrated	17 (25.37)
When I am with my friends	17 (25.37)
When I drink coffee or tea	16 (23.88)
When I feel depressed	9 (13.43)
When someone offers me a cigarette	6 (8.96)
When I feel awkward when with others	5 (7.46)
When I see someone enjoying a cigarette	4 (5.97)
When I gain weight	1 (1.49)
**Categories of personalized alternative activities**	**Participants who provided the category of personalized alternative activities: *n* (%)**
Eating (e.g., food, fruits, sweets, and snack)	55 (82.09)
Drinking (e.g., tea, water, milk, and coffee)	48 (71.64)
Participating in social activities with non-smokers (e.g., talking, joking, and consulting)	48 (71.64)
Walking around, strolling, or hiking (e.g., getting fresh air)	48 (71.64)
Doing sports (e.g., running, biking), mediation, yoga, or breathing exercises	36 (53.73)
Reading, writing, or drawing (e.g., reading books, keeping diary, and making plans)	31 (46.27)
Going to toilet (e.g., taking a shower, brushing teeth, making up, and playing phone)	31 (46.27)
Seeking help or support (e.g., calling or sending text messages to others)	30 (44.78)
Taking chewing gum or lollipop	28 (41.79)
Participating in music-related activities (e.g., listening, playing, and dancing)	26 (38.81)
Saying “no” to others or myself or walking away from smokers	26 (38.81)
Doing housework (e.g., cleaning, packing, and working in the garden)	24 (35.82)
Browsing social media (e.g., Facebook and Instagram) by phone or computer	23 (34.33)
Playing games (e.g., mobile or computer games, cards, billiards, and chess)	21 (31.34)
Staying inside in the public area (e.g., office and restaurant)	20 (29.85)
Watching TV or movie	16 (23.88)
Cognitive coping (e.g., thinking advantages and disadvantages of smoking, reminding plans for quit smoking, and self-affirmation)	13 (19.40)
Keeping hands busy (e.g., doing handwork or holding objects)	10 (14.93)
Hanging on the couch or taking a nap	8 (11.94)
Going shopping	6 (8.96)
Playing with family members (e.g., children or pets)	6 (8.96)
Others	16 (23.88)

**Table 2:** Baseline characteristics

	ApBM + (*n* = 26)	Standard-ApBM (*n* = 19)	Sham-ApBM (*n* = 22)	*F*(2, 64)/*χ*^2^(2)	*p*-value
**Demographics**					
Age (years) * M* (*SD*)	30.19 (13.62)	27.42 (10.95)	29.77 (11.97)	0.30	0.740
Gender, n (%)				0.92	0.633
Male	9 (34.6)	9 (47.4)	10 (45.5)	-	-
Female	17 (65.4)	10 (52.6)	12 (54.5)	-	-
Education, n (%)				0.34	0.845
≥ Bachelor	18 (69.2)	12 (63.2)	15 (71.4)	-	-
< Bachelor	8 (30.8)	7 (36.8)	6 (28.6)	-	-
**Smoking-related variables**					
Duration of smoking (years) * M* (*SD*)	13.35 (13.40)	10.76 (10.91)	12.02 (8.73)	0.29	0.751
Daily cigarette consumption (in the past half-year) * M* (*SD*)	12.77 (6.29)	15.00 (5.98)	13.86 (5.07)	0.81	0.450
FTND (0–10) * M* (*SD*)	3.35 (2.24)	3.58 (2.36)	4.09 (2.29)	0.64	0.528
Previous quit attempts (times) * M* (*SD*)	3.19 (2.76)	4.37 (4.40)	3.50 (4.11)	0.56	0.573
RCQ (-24–24) * M* (*SD*)	7.12 (5.90)	9.63 (5.43)	9.64 (4.39)	1.79	0.175

*ApBM*, Approach Bias Modification; *FTND*, Fagerström Test for Nicotine Dependence; *RCQ*, Readiness to Change Questionnaire

**Table 3:** Summary statistics on outcomes^a^ by Condition and Time

Outcomes/Time	ApBM +	Standard-ApBM	Sham-ApBM	*F*(2, 64)	*p*-value
**ApB for smoking,** ***M*** (***SD***)					
Pre-test	16.41 (69.71)	10.76 (30.98)	7.89 (58.20)	0.14	0.871
Post-test	− 24.36 (69.19)	− 16.86 (32.60)	− 9.27 (63.73)	-	-
1-month follow-up	− 5.83 (88.46)	− 6.65 (45.47)	2.93 (51.84)	-	-
**ApB for alternative activities,** ***M*** (***SD***)					
Pre-test	− 2.67 (57.88)	14.84 (48.84)	− 9.31 (54.85)	1.06	0.353
Post-test	− 17.19 (66.42)	− 4.15 (43.26)	8.34 (62.55)	-	-
1-month follow-up	4.45 (37.62)	− 7.01 (34.00)	6.45 (49.94)	-	-
**ApB for neutral activities,** ***M*** (***SD***)					
Pre-test	− 1.91 (58.79)	8.31 (56.47)	3.45 (43.00)	0.20	0.816
Post-test	− 14.01 (49.76)	− 16.79 (27.73)	5.51 (81.01)	-	-
1-month follow-up	9.80 (33.05)	− 4.55 (39.51)	3.92 (56.28)	-	-
**QSU,** ***M*** (***SD***)					
Pre-test	25.08 (7.89)	29.79 (10.13)	26.00 (7.79)	1.78	0.176
Post-test	16.12 (7.70)	20.83 (10.92)	17.14 (7.88)	-	-
1-month follow-up	12.50 (3.15)	17.18 (10.45)	17.71 (7.30)	-	-
**DCC,** ***M*** (***SD***)					
Pre-test	13.14 (7.28)	13.92 (5.65)	13.68 (5.05)	0.10	0.910
Post-test	4.07 (6.48)	3.77 (6.31)	3.21 (4.92)	-	-
1-month follow-up	3.48 (3.65)	3.98 (5.99)	5.70 (6.33)	-	-
**CO levels,** ***M*** (***SD***)					
Pre-test	13.65 (8.21)	13.79 (6.05)	14.77 (7.47)	0.15	0.858
Post-test	5.80 (7.14)	6.74 (7.24)	5.80 (5.25)	-	-
1-month follow-up	4.35 (3.08)	5.06 (4.59)	7.25 (6.77)	-	-
**7D-PPA, *n***_1_/***n***_2_^b^ (*%*)_1_					
Pre-test	0 (0.00)	0 (0.00)	0 (0.00)	-	-
Post-test	7/26 (26.9)	5/19 (26.3)	5/22 (22.7)	-	-
1-month follow-up	4/18 (22.2)	4/17 (23.5)	4/21 (19.0)	-	-

*ApBM*, Approach Bias Modification; *ApB*, approach biases; *QSU*, Questionnaire on Smoking Urge; *DCC*, daily cigarette consumption; *CO levels*, breath carbon monoxide level; *7D-PPA*, 7-day point prevalence abstinence. ^a^The exact number of participants who completed each outcome at post-test and 1-month follow-up can be found in the participant flowchart (see Fig. S1 in Electronic Supplementary Material 1). ^b^The number of participants coded as 1 (i.e., quit) at a test time-point divided by the total number of participants who reported their smoking status at the same test time-point

On average, participants spent 3.12 weeks (*SD* = 1.26) participating in the whole intervention, which did not differ across the three training conditions (*F*(2, 64) = 2.07, *p* = 0.135). All participants received the brief motivational smoking intervention and an average of 4.31 sessions of ApBM training (*SD* = 0.94), which did not differ across the three training conditions as well (*F*(2, 64) = 1.87, *p* = 0.163).

### Craving Manipulation Check

We first conducted a quality check of the mental imagery procedure (MIP) on two outcomes. First, across training sessions and regardless of training conditions, over 95% of participants provided relevant texts to describe the situations they had chosen, read, and imagined in the MIP, confirming that participants had followed the instructions of the MIP. Second, based on the results of one-way repeated ANOVAs, the vividness scores participants rated for their image during the MIP did not differ across training sessions in all conditions (*p*s ≥ 0.254); and based on the results of a one-way ANOVA, the average vividness scores across training sessions did not differ between training conditions as well (*F*(2, 63) = 2.11; *p* = 0.130). Hence, across training sessions and regardless of training conditions, participants had similar engagement levels during the MIP (vividness scores: *M* = 57.37, *SD* = 16.34; ranged from 0 to 100).

To test whether MIP successfully increased craving for the ApBM + condition, several one-way repeated ANOVAs (for training session effect) and a one-way ANOVA (for condition effect) were conducted. Results showed that craving changes from before to after MIP did not differ across training sessions in all training conditions (*p*s ≥ 0.135), but the average changes of craving from before to after MIP across training sessions differed within training conditions (*F*(2, 63) = 8.96; *p* < 0.001). Pairwise comparisons showed that in the ApBM + condition, craving increased significantly from before MIP (*M* = 32.51, *SD* = 16.04) to after MIP (*M* = 36.42, *SD* = 14.50; *t*(24) =  − 2.64, *p* = 0.014, *d* = 0.54), whereas in the standard-ApBM condition, craving decreased significantly from before MIP (*M* = 37.79, *SD* = 18.87) to after MIP (*M* = 33.72, *SD* = 19.22; *t*(18) = 2.35, *p* = 0.031, *d* = 0.54), and the same pattern of decrease was found for the sham-ApBM condition from before MIP (*M* = 32.98, *SD* = 13.93) to after MIP (*M* = 25.20, *SD* = 13.53; *t*(21) = 2.77, *p* = 0.011, *d* = 0.59). Thus, as we expected, the MIP increased craving in the ApBM + condition only. However, it should be noted that, after the MIP, the craving levels in the ApBM + condition were only significantly higher than those in the sham-ApBM condition (*M*_*diff*_ = 11.23, *p* = 0.017, *d* = 0.80) and did not differ from those in the standard-ApBM condition (*M*_*diff*_ = 2.71, *p* = 0.573, *d* = 0.16). Moreover, after the MIP, the craving levels did not differ between standard-ApBM and sham-ApBM conditions (*M*_*diff*_ = 8.52, *p* = 0.088, *d* = 0.52).

### Training Effects at Post-Test

#### Primary Outcomes: Approach Biases

Results showed a main effect of Time in reducing ApB for smoking pictures from pre-test to post-test (*F*(1,63) = 10.37, *p* = 0.002, η_p_^2^ = 0.141), but no interaction effect by Condition (*F*(2,63) = 0.95, *p* = 0.394, η_p_^2^ = 0.029; see Table 3). Regarding changes of ApB for alternative-activity pictures and for neutral-activity pictures from pre-test to post-test, there was neither a significant effect of Time (alternative-activity pictures: *F*(1,63) = 0.41, *p* = 0.524, η_p_^2^ = 0.006; neutral-activity pictures: *F*(1,63) = 1.40, *p* = 0.240, η_p_^2^ = 0.022) nor any significant interactions with Condition (alternative-activity pictures: *F*(2,63) = 1.57, *p* = 0.217, η_p_^2^ = 0.047; neutral-activity pictures: *F*(2,63) = 0.89, *p* = 0.416, η_p_^2^ = 0.027; see Table 3).

**Fig. 1:**
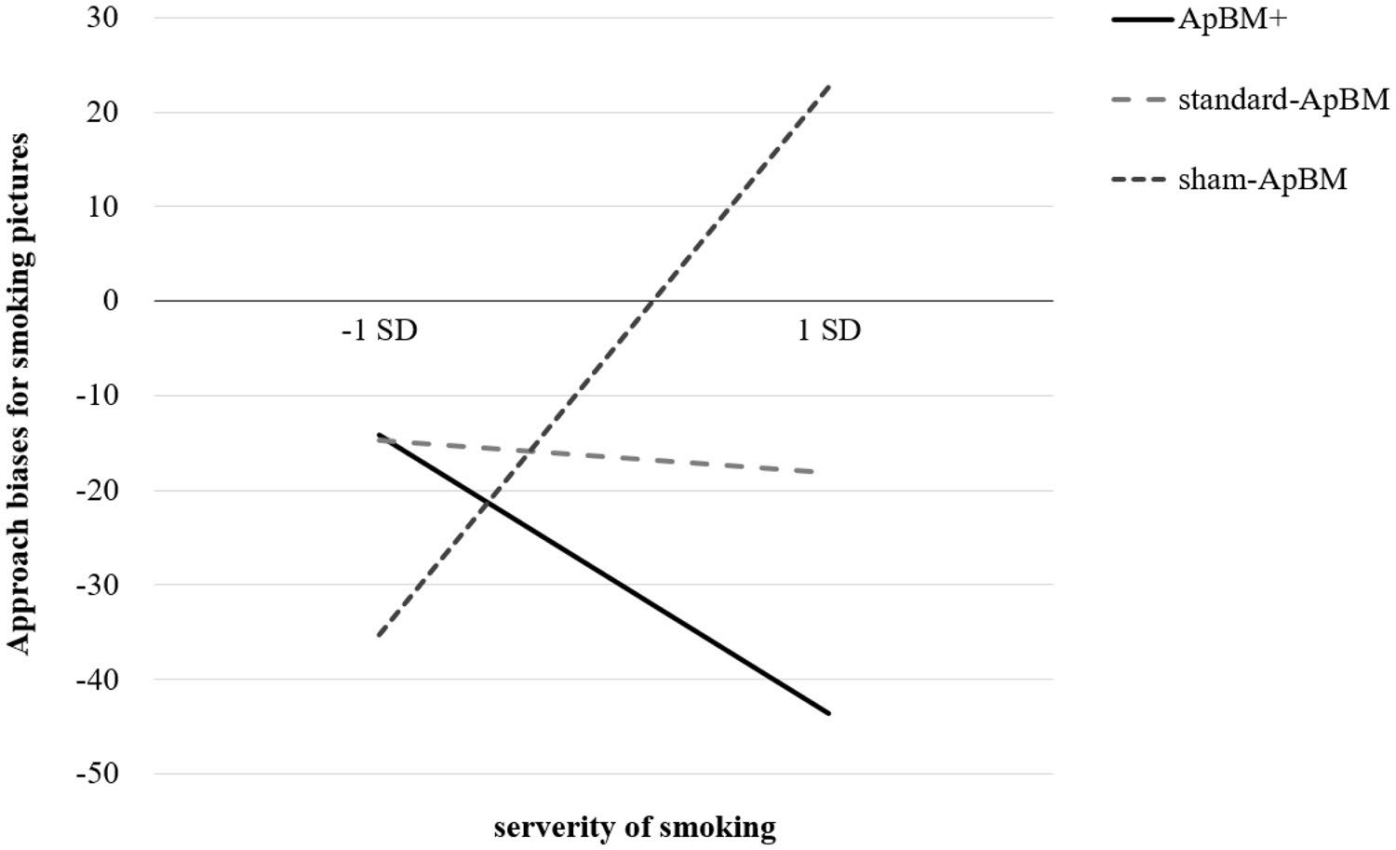
The interaction between Condition and severity of smoking (− 1 SD vs. + 1 SD) in predicting approach biases for smoking pictures (higher index indicates stronger biases) at post-test. *Note.* ApBM, Approach Bias Modification

Moderation analyses revealed an interaction effect of Condition and severity of smoking at a statistical trend level regarding changes of ApB for smoking pictures (*F*(2,59) = 3.13, *p* = 0.051, η_p_^2^ = 0.096). This effect was not found for changes of ApB for alternative-activity or neutral-activity pictures (*p*s ≥ 0.247). Specifically, from pre-test to post-test, ApBM + was more effective in reducing ApB for smoking than sham-ApBM in relatively heavy smokers (*b* =  − 43.65, *95% CI* = [− 78.62, − 8.67], *p* = 0.015, η_p_^2^ = 0.096), but this difference was not found between ApBM + and standard-ApBM (*b* =  − 12.96, *95% CI* = [− 45.48, 19.55], *p* = 0.428, η_p_^2^ = 0.011), or between standard-ApBM and sham-ApBM (*b* =  − 30.68, *95% CI* = [− 68.72, 7.36], *p* = 0.112, η_p_^2^ = 0.042; see Fig. 1). When considering the simple slopes (see Fig. 1), it is noteworthy to mention, first, that after receiving sham-ApBM, the ApB for smoking increased in heavy smokers, but it decreased in light smokers (*b* = 28.95, *95% CI* = [0.82, 57.08], *p* = 0.044, η_p_^2^ = 0.067); second, after receiving ApBM + , the ApB for smoking reduced more in heavy smokers than in light smokers (*b* =  − 14.69, *95% CI* = [− 35.27, 5.89], *p* = 0.158, η_p_^2^ = 0.033), but this latter effect was not significant.

#### Secondary Outcomes: Smoking-Related Behaviors

Results showed a main effect of Time in reducing craving (*F*(1,62) = 46.75, *p* < 0.001, η_p_^2^ = 0.430), daily cigarette consumption (*F*(1,62) = 124.64, *p* < 0.001, η_p_^2^ = 0.668), and CO levels (*F*(1,61) = 69.66, *p* < 0.001, η_p_^2^ = 0.533) from pre-test to post-test, in the absence of interactions with Condition (*p*s ≥ 0.868; see Table 3). The 7-day point prevalence abstinence rates at post-test did not differ across the three training conditions (χ^2^ (2) = 0.12, *p* = 0.940, Cramer’s V = 0.043; see Table 3). Additionally, moderation analyses showed no significant interaction effects of Condition and severity of smoking on all smoking-related behavior outcomes at post-test (*p*s ≥ 0.473).

### Training Effects at 1-Month Follow-up

The results demonstrated no interactions between Condition and Time on changing ApB, craving, daily cigarette consumption, and CO levels at 1-month follow-up; and 7-day point prevalence abstinence rates at 1-month follow-up did not differ across the three training conditions. There was only a main effect of Time in reducing craving, daily cigarette consumption, and CO levels from pre-test to 1-month follow-up (see Table 3). Additionally, moderation analyses showed no significant interaction effects of Condition and severity of smoking on all outcomes at 1-month follow-up.

## Discussion

The goal of this pilot study was to test a new variety of ApBM (ApBM +): in a context of increased craving, participants were trained to not only avoid smoking but also to approach personalized behavioral alternative activities for smoking. These findings indicate that generally, ApBM + was not superior to standard-ApBM and sham-ApBM in changing approach biases and smoking-related behaviors. There was some indication of a difference in changes of smoking-approach biases between sham-ApBM and ApBM + in relatively heavy smokers at post-test. However, this was primarily driven by a significant increase in smoking-approach biases in the sham-ApBM and a trend of decrease in smoking-approach biases in the ApBM + .

The lack of differential changes in approach biases and smoking behaviors across the whole sample adds to previous inconsistent findings regarding the effects of ApBM within the smoking literature [4, 12–18]. The current results revealed a general reduction in smoking-approach biases from pre-test to post-test and smoking behaviors over time in all three ApBM conditions. There are several possible explanations for the main effect of time. First, using 50% smoking avoidance pictures in the sham-ApBM could be regarded as a minimal-dose intervention [42–44]. Second, all participants were exposed to their self-selected alternative actives for smoking in the ApBM training regardless of training condition. Although this design is “clean” from a methodological perspective, simply seeing the pictures and approaching them 50% of the time may have been sufficient to exert a positive effect. Third, it is possible that the training sessions were ineffective and that participants benefited solely from the brief motivational smoking intervention. Future studies could include a treatment-as-usual condition to compare the addition of ApBM.

In ApBM + , we replaced the neutral stimuli (often office supplies) with representations of personalized behavioral alternative activities for smoking as the approach category. By doing so, we aimed to increase participants’ approach biases for alternative activities. But our results did not support such notions; instead, ApBM + demonstrated no differences among conditions in changing such approach biases. This result was at odds with two recent studies in the smoking field that similarly trained smokers to approach positive stimuli or meaningful alternative activities stimuli. In one study, an approach response to positive stimuli was demonstrated [4], while the second study showed an increased association between negative emotion and alternative activities for smoking [18]. We note that the former study used standardized approach-stimuli for everyone, and although the latter study used personalized alternative activities, these stimuli were also standardized by the researchers. In our study, we introduced self-selected personalized stimuli reflecting real-life scenes and activities. As a result, the approach-stimuli we used were more complex and diverse, which may have negatively affected the reliability of our assessment [45, 46]. Future research on tailored ApBM training may benefit from controlling for stimuli complexity by instructing participants to select alternative activities (perhaps as well as smoking and neutral stimuli) from a large pre-selected and structured pool of stimuli. Regarding the alternative activities, eating and drinking healthy food and drinks, participating in social activities with non-smokers, and doing sports might be the focus in further studies, as smokers might benefit from naturally rewarding properties of those stimuli [47] and they were top alternative activities selected by the smokers in the current study (see Table 1).

Additionally, in ApBM + , mental imagery scripts of participants’ personal smoking risk situations were used to increase participants’ craving before the training. Thus, participants were expected to conduct the training in the context of cue-evoked craving, with the aim to help transfer training effects to real-life situations. We indeed observed that craving significantly increased from before to after the MIP in ApBM + only; however, the effect was moderate (from a score of 32.51 to 36.42 (the range of the craving is 0–100), *d* = 0.54). Likewise, after the MIP, the craving levels were still moderate in the ApBM + , with only a significant difference between ApBM + and sham-ApBM (*M*_diff_ = 11.23), and no difference between ApBM + and standard-ApBM (*M*_diff_ = 2.71). Taken together, it is questionable whether this moderate increase in craving was sufficient to create a “hot” context and foster the desired transfer of training effects to real-life risk situations, and whether the small difference of craving between conditions produced by MIP could lead to different training effects. Given that previous cue-reactivity research showed that mental imagery scripts usually increase craving for smoking with a large effect size of *d* = 1.18 [38], deemed necessary for cue exposure interventions to be effective [48]. Thus, the current craving induction may not have been sufficiently strong enough, perhaps related to the relatively low vividness scores of participants’ mental imagery of smoking risk situations [49]. Stronger manipulations should preferably be tested, especially within a home setting, for example, using filmed presentations of smoking-related cues or video monitor simulations of multiple real-word environments with smoking-related cues [50, 51]. Additionally, further research could investigate whether instructing participants to conduct the ApBM when experiencing craving in their daily lives could improve the ApBM effects (e.g., via mobile-based just-in-time interventions [52]).

Our exploratory moderation analyses suggested that sham-ApBM training may not be an appropriate control condition to apply for relatively heavy smokers, as we observed an increase in smoking-approach biases, adding to the current discussions regarding control conditions in CBM [42, 43]. However, a positive effect in reducing smoking-approach biases in relatively heavy smokers was indicated for the ApBM + condition, despite not reaching a significance level. This divergent training effect in relatively heavy smokers is difficult to explain based on our current design. One possible explanation could be that in comparison to light smokers, heavy smokers may have generally higher levels of craving, forming the foundation of the training context, regardless of their training condition. In this way, the higher state of craving may lead relatively heavy smokers to benefit from ApBM + but got worse from sham-ApBM. Our additional exploratory analyses showed that descriptively, heavy smokers indeed showed higher levels of craving when compared to light smokers in ApBM + and sham-ApBM before conducting the training (details can be found in Electronic Supplementary Material 7). Additionally, in line with our hypotheses, the indication of a more positive effect of ApBM + in reducing smoking-approach biases in relatively heavy smokers would have been related to an increase in approach biases for alternative activities in this subgroup. However, our moderation analysis on approach biases for alternative activities did not find evidence for such an association, and they may be related to a combination of power issues and low assessment reliabilities.

Moreover, despite our moderation analyses showing an indication of a divergent training effect in changing smoking-approach biases in relatively heavy smokers, the effect did not transfer to smoking behaviors. These results seem to be at odds with recent reviews on CBM that demonstrate changes in cognitive biases are often related to changes of substance use outcomes [7, 42]. However, the potential indirect effects of the training via changes of smoking-approach biases on changing smoking behaviors should not be discounted. To investigate such indirect effects, a series of moderated mediation analyses could be conducted in future research with a larger sample size. Although the moderation results discussed above are preliminary, they can help guide future improvements to training varieties. In light of contradictory results of CBM in smoking, recent papers have questioned the interventions underlying mechanisms. For example, ApBM might achieve positive effects by modifying (automatically activated) inferences regarding consequences of stimulus-driven actions rather than associations [53]. Based on this idea, a novel consequence-based ApBM training was developed and tested within the eating domain, with promising effects [54]. Importantly, in each trial, participants make a decision (i.e., approach or avoid healthy food), after which they are shown the consequences of their choice (i.e., see a healthy or sick body of an avatar representing themselves), which differs from consistently training avoidance as in current ApBM. In addition, personalized alternative activities (as tested here and in Kopetz et al. [18]) and personalized risk contexts can be included in the consequence-based ApBM training as was recently proposed [55]. These new theory-based varieties await empirical testing.

This pilot study has several limitations. First, while statistical power for the primary outcomes at post-test was reasonable, it was low for detecting short-term follow-up effects. Second, the reliability of AAT was low, perhaps related to its application as an online assessment, combined with the possible limitations resulting from using keys instead of a joystick. Although a zoom feature was incorporated within the keyboard version such that pushing and pulling was clearly associated with approach (i.e., picture size increases) and avoidance (i.e., picture size increases) — comparable to the joystick version, no study has yet directly compared the ApBM effects with the different operational methods. In fact, the low reliability of AAT was not an exception in this study; it was consistent with most implicit reaction time tasks [45, 46]. By using a task with such low reliability, it remains difficult to assess any training-induced changes of cognitive biases reliably. Therefore, generally speaking, in order to boost CBM research, it is necessary to develop more reliable experimental tasks for measuring cognitive biases in the future. Third, not all participants in this study completed all training sessions (i.e., seven sessions), which may have hindered detecting potential training effects. Fourth, two possible improvements (i.e., training smokers to approach alternative activities for smoking and training smokers in a state of craving) were added into ApBM + (as is often the case in an intervention package), which is not ideal from an experimental design, as we cannot distinguish their unique effects.

In conclusion, the current ApBM + version did not improve the effectiveness of ApBM in changing approach biases and smoking outcomes. The indication of divergent training effects in changing smoking-approach biases between sham-ApBM and ApBM + in relatively heavy smokers may warrant further research with a larger sample size. Specifically, the unexpected increase in smoking-approach biases after sham-ApBM in relatively heavy smokers brings questionable doubt as to the appropriateness of the sham control condition in this subgroup. A control condition which would not affect relatively automatic cognitive-motivational processes is recommended for future research (e.g., treatment-as-usual [42, 43]). Additionally, a trend of decrease in smoking-approach biases after ApBM + in relatively heavy smokers pointed towards an improved ApBM training variety. The current ApBM + version may be improved by (1) creating a better (i.e., more real) personalized smoking-related context as a training context, (2) training using personalized but less complex stimuli to improve the reliabilities of the tasks for measuring cognitive biases, and (3) introducing personally relevant consequences to reinforce the newly learned responses (e.g., linking a healthy body to smoking-avoidance responses and alternative activities-approach responses and linking a sick body to smoking-approach responses and alternative activities-avoid responses [54, 55]). For this suggested ApBM variety, it is also interesting and important to investigate what are its potential working mechanisms that may lead to changes of behaviors, via such as changes of cognitive biases, changes of inferences regarding consequences of stimulus-driven actions, or both.

## Supplementary Information

Below is the link to the electronic supplementary material.

Supplementary file1 (DOC 5783 KB)
