# Supplementary material for: A Pilot Study on Approach Bias Modification in Smoking Cessation: Activating Personalized Alternative Activities for Smoking in the Context of Increased Craving
Source: Int J Behav Med. 2021 Oct 25;29(4):480–93. doi: 10.1007/s12529-021-10033-x (PMC9338119; doi:10.1007/s12529-021-10033-x)
Supplement: Supplementary file 1 — Supplementary file1 (DOC 5783 KB) [file 12529_2021_10033_MOESM1_ESM.doc]

**Electronic Supplementary Materials**

**A Pilot Study on Approach Bias Modification in Smoking Cessation: Activating Personalized Alternative Activities for Smoking in the Context of Increased Craving:**

Si Wen^1^, MEd; Helle Larsen^1,2^, PhD; Reinout W. Wiers^1,3^, PhD

^1^ Addiction Development and Psychopathology (ADAPT)-lab, Department of Developmental Psychology, University of Amsterdam, Amsterdam, the Netherlands

^2^ Research Priority Area Yield, University of Amsterdam, Amsterdam, the Netherlands

^3^ Center for Urban Mental Health, University of Amsterdam, Amsterdam, the Netherlands

**Authors’ contact information:**

*Si Wen* (corresponding author), Mailing address: Department of Developmental Psychology, University of Amsterdam, Postbus 15916, 1001 NK Amsterdam, the Netherlands; E-mail: si-wen@hotmail.com; ORCID: https://orcid.org/0000-0003-1152-2608.

*Helle Larsen*, E-mail: H.Larsen@uva.nl; ORCID: https://orcid.org/0000-0003-0117-9252.

*Reinout W. Wiers*, E-mail: R.W.H.J.Wiers@uva.nl; ORCID: https://orcid.org/0000-0002-4312-9766.

**Electronic Supplementary Material 1:**

**
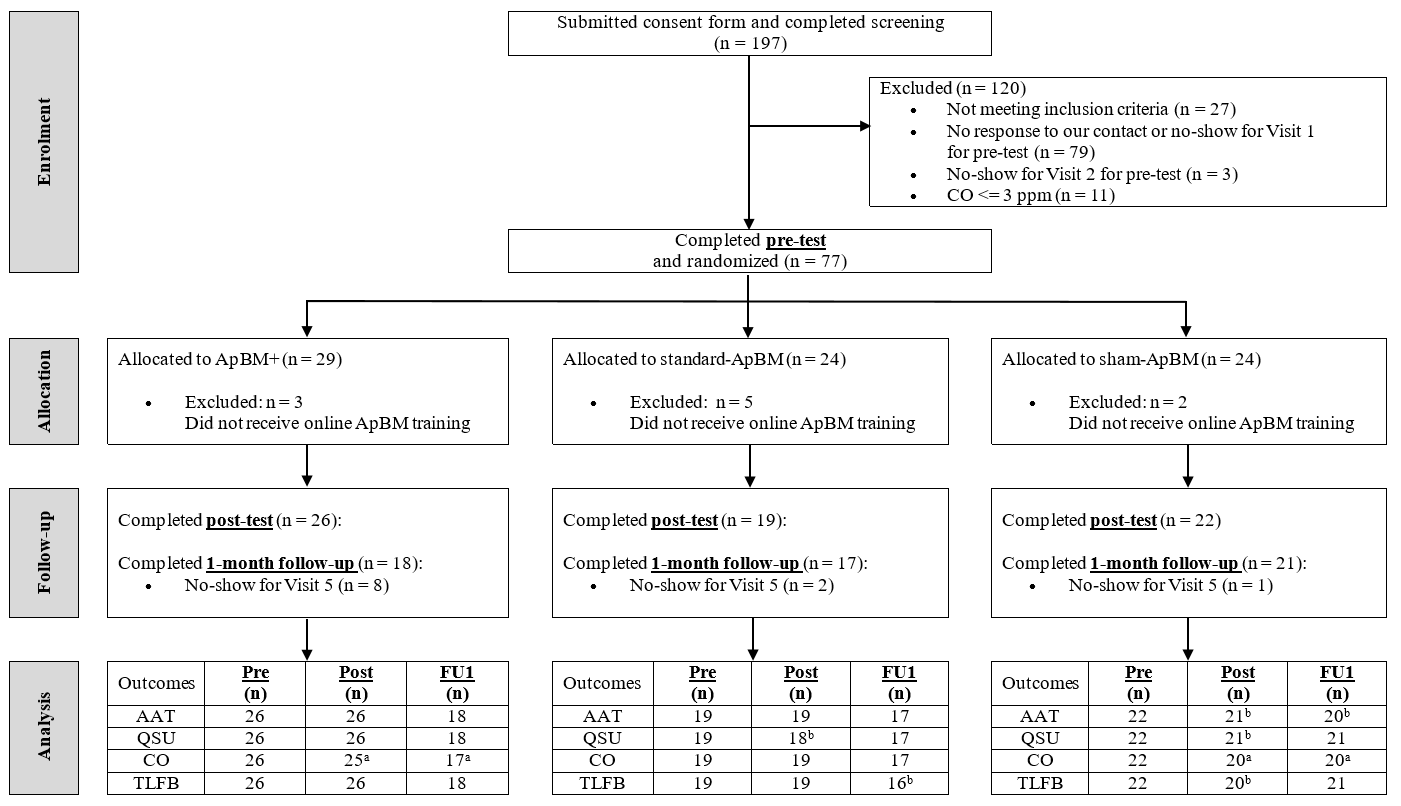
**

**Fig. S1** Participant flowchart.

*Note.* ApBM = Approach Bias Modification; AAT = Approach-Avoidance Task; QSU = Questionnaire on Smoking Urge; CO = breath carbon monoxide level; TLFB =

TimeLine Follow Back; Pre = pre-test; Post = post-test; FU1 = 1-month follow-up. Missing data: ^a^participants did not attend the lab but completed the assessment at home;

thus, we were not able to collect their CO levels; ^b^participants finished the tests, but the data were not correctly stored due to a technical error.

**Electronic Supplementary Material 2: Split-half reliability estimates of the Approach-Avoidance Task**

Bootstrapped split-half reliability estimates [1] for each picture category of the Approach-Avoidance Task (AAT) were obtained by using the *splithalf* package in R (version 0.5.2 [2]), which performed 5000 random splits. Reliability of the AAT was *r* = 0.25, *95% CI* = [0.09, 0.41] for smoking pictures, *r* = 0.23, *95% CI* = [0.06, 0.39] for alternative-activity pictures, and *r* = 0.23, *95% CI* = [0.05, 0.39] for neutral-activity pictures (Spearman-Brown corrected *r*_sb_ = 0.39, 95% *CI* = [0.16, 0.59], *r*_sb_ = 0.36, 95% *CI* = [0.12, 0.56], and *r*_sb_ = 0.36, 95% *CI* = [0.10, 0.57] for smoking, alternative-activity, and neutral-activity pictures, respectively). The reliabilities of the AAT for each picture category was fairly poor, but was in line with previous studies [3–5] and was not unusual for tests assessing cognitive biases using reaction-time based measurements [6,7].

**Electronic Supplementary Material 3: Selection criteria of personalized alternative activities pictures**

Participants searched and provided their alternative activities pictures according to the following criteria: (1) they were allowed to provide repeated alternative activities for different risk situations, as long as the alternative activities were meaningful to them, (2) they were encouraged to search for different pictures even for the same alternative activity (e.g., running with friends or running with a dog); and (3) all pictures were required to represent active rather than passive contents (e.g., someone drinking water rather than a static glass of water).

**Electronic Supplementary Material 4: Randomization and blinding**

A computer program developed by the University of ([omitted for blind review]), named “Lotus” was used to randomize participants. The precise algorithm of the randomization stratified participants by gender and daily cigarette consumption in the past half-year with a 1:1:1 ratio; therefore, participants were randomly assigned to one of the three ApBM training conditions to which the fewest participants of their gender and severity of smoking had been allocated so far. The automatic randomization procedure ensured the allocation concealment. Both participants and researchers were blinded to the experimental conditions participants were assigned to.

**Electronic Supplementary Material 5:**

**
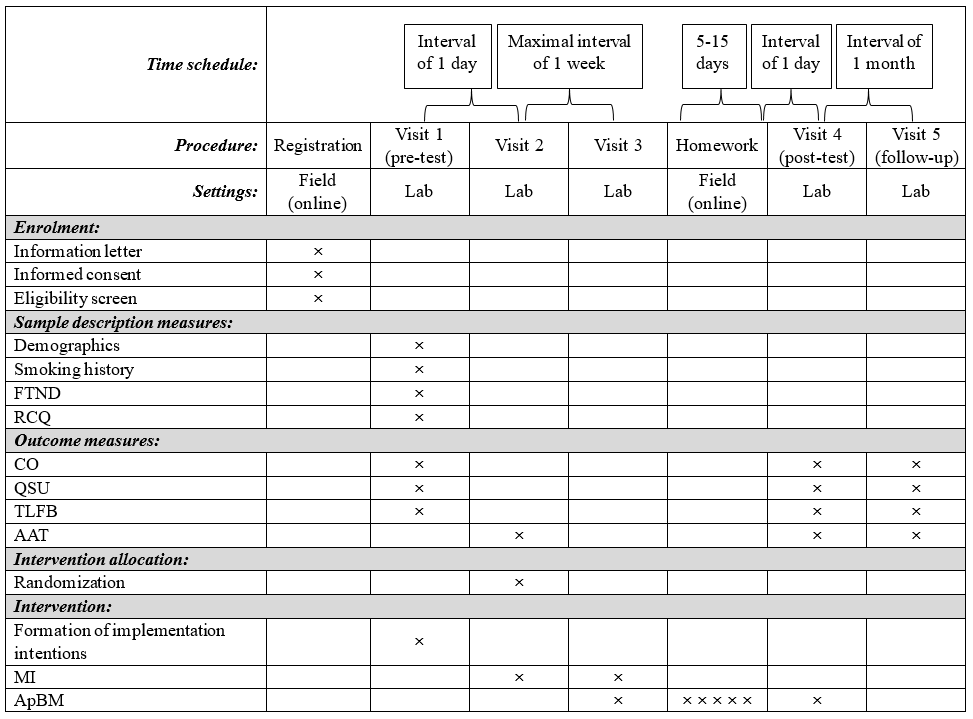
**

**Fig. S2** Study procedure.

*Note.* FTND = Fagerström Test for Nicotine Dependence; RCQ = Readiness to Change Questionnaire; CO = breath carbon monoxide level; QSU = Questionnaire on Smoking Urge; TLFB = TimeLine Follow Back; AAT = Approach-Avoidance Task; MI: Motivational Interviewing; ApBM = Approach Bias Modification.

**Electronic Supplementary Material 6: Intention-to-treat analyses**

**Methods**

Training effects at both post-test and 1-month follow-up were also tested using an intention-to-treat (ITT) analysis method as a sensitivity analysis. The ITT analyses were conducted based on the randomized sample (*N* = 77; see Fig. S1 for participant flowchart in Electronic Supplementary Material 1). The percentage of missing data reflected13.0% of outcomes measured at post-test and 27.3% of outcomes measured at 1-month follow-up. Prior to the ITT analyses, missing data handled by performing multiple imputations, using the Predictive Mean Matching (PMM) method in SPSS [8], creating 50 imputed data sets.

In this study, the imputation models were built based on two principles [9]: (1) the imputation models must be at least as complex as the analysis models, and (2) auxiliary variables were suggested to be included in the imputation models to enhance the plausibility of missing at random (MRA) assumptions. Based on the first principle, the models used to explore the moderation effects examining the influence of smoking severity on the relationship between training effects and the study outcomes were carried forward and treated as the basic foundation of the imputation models. That is, within the imputation model, we entered the experimental condition (Condition), severity of smoking, and their interaction term as predictors for each outcome. Further, we also entered the Z-standardized pre-test value of that outcome as a predictor when the outcome was continuous. Additionally, based on the second principle, baseline variables with high missing value probabilities and baseline variables that differed between intervention conditions should be included within the imputation models as auxiliary variables. Therefore, we compared the observed baseline variables between training completers and training dropouts and the observed baseline variables across training conditions.

Results showed that (1) compared to the training completers at post-test (*n* = 67), the training dropouts (*n* = 10) were older, smoked for relatively more years, reported higher scores in readiness to change, and showed more avoidance biases for both smoking pictures and neutral-activity pictures (age: *M_dropouts_* = 37.80, *SD* = 12.48, *M_completers_* = 29.27, *SD* = 12.25, *t*(75) = 2.05, *p* = 0.044; duration of years of smoking: *M_dropouts_* = 22.70, *SD* = 12.32, *M_completers_* = 12.18, *SD* = 11.22, *t*(75) = 2.73, *p* = 0.008; readiness to change: *M_dropouts_* = 14.00, *SD* = 4.97, *M_completers_* = 8.66, *SD* = 5.38, *t*(75) = 2.96, *p* = 0.004; ApB for smoking: *M_dropouts_* = -40.00, *SD* = 70.63, *M_completers_* = 12.01, *SD* = 56.51, *t*(75) = -2.63, *p* = 0.010; ApB for neutral-activity: *M_dropouts_* = -33.69, *SD* = 40.00, *M_completers_* = 2.75, *SD* = 52.77, *t*(75) = -2.09, *p* = 0.040); (2) compared to the training completers at 1-month follow-up (n = 56), the training dropouts (n = 21) smoked for relatively more years and reported higher scores on nicotine dependence (duration of years of smoking: *M_dropouts_* = 19.10, *SD* = 12.22, *M_completers_* = 11.46, *SD* = 11.09, *t*(75) = 2.62, *p* = 0.011; nicotine dependence: *M_dropouts_* = 4.76, *SD* = 2.21, *M_completers_* = 3.48, *SD* = 2.19, *t*(75) = 2.28, *p* = 0.026); and (3) there were no significant baseline differences in participants’ characteristics and training outcomes measured at pre-test across the three training conditions (see Tables S1 and S2). Based on these results, participants’ age, duration of years of smoking, nicotine dependence, readiness to change, and their approach biases for both smoking pictures and neutral-activity pictures measures at pre-test were deemed necessary for inclusion within the imputation models as auxiliary variables, as the missing data depended on these observed variables. However, participants’ age and duration of years of smoking were highly correlated (*r* = 0.92, *p* < 0.001), as were nicotine dependence and severity of smoking (*r* = 0.72, *p* < 0.001). In order to avoid problems with collinearity, age and nicotine dependence were not entered in the imputation models. As a result, duration of years of smoking, readiness to change, and approach biases for smoking and neutral-activity pictures measured at pre-test were entered for each imputation model as auxiliary variables.

To test the training effects on the primary and secondary outcomes, and to explore the moderation effects of severity of smoking in the relationship between training effects and all outcomes, analyses used in the complete case analyses were applied to the ITT analyses (details were described in the section of Statistical Analyses in the main manuscript). All analyses were conducted within the 50 multiple imputation data sets, and the results were pooled. SPSS does not automatically provide the pooled results for any ANOVA [10]; therefore, the results (i.e., omnibus effects of the training effects) were pooled either following an SPSS syntax for mixed-model ANOVA [11] or an ad hoc method for regression analyses (i.e., averaging the *F*-values across multiply imputed data sets [12]).

**Table S1 Baseline characteristics in ITT sample (*N* = 77)**

|  | ApBM+  (*n* = 29) | Standard-ApBM  (*n* = 24) | Sham-ApBM  (*n* = 24) | *F*(2, 74)  /*χ*^2^(2) | *p-value* |
| --- | --- | --- | --- | --- | --- |
| ***Demographics*** |  |  |  |  |  |
| Age (years)  *M* (*SD*) | 32.00 (14.30) | 29.50 (11.43) | 29.29 (11.57) | 0.39 | 0.681 |
| Gender, n (%) |  |  |  | 1.99 | 0.369 |
| Male | 9 (31.0) | 12 (50.0) | 10 (41.7) | - | - |
| Female | 20 (69.0) | 12 (50.0) | 14 (58.3) | - | - |
| Education, n (%) |  |  |  | 0.97 | 0.616 |
| ≥ Bachelor | 18 (62.1) | 15 (62.5) | 17 (73.9) | - | - |
| < Bachelor | 11 (37.9) | 9 (37.5) | 6 (26.1) | - | - |
| ***Smoking-related variables*** |  |  |  |  |  |
| Duration of smoking (years)  *M* (*SD*) | 15.28 (14.24) | 13.31 (11.71) | 11.69 (8.42) | 0.61 | 0.549 |
| Daily cigarette consumption  (in the past half-year)  *M* (*SD*) | 13.24 (6.37) | 15.71 (7.07) | 14.08 (4.96) | 1.05 | 0.354 |
| FTND (0-10)  *M* (*SD*) | 3.59 (2.24) | 3.83 (2.35) | 4.13 (2.23) | 0.37 | 0.693 |
| Previous quit attempts (times)  *M* (*SD*) | 3.24 (2.63) | 4.67 (4.51) | 3.50 (3.96) | 1.06 | 0.353 |
| RCQ (-24-24)  *M* (*SD*) | 7.76 (5.96) | 10.50 (5.88) | 10.13 (4.51) | 1.96 | 0.148 |

*Note.* ITT = intention-to-treat analyses. ApBM = Approach Bias Modification; FTND = Fagerström

Test for Nicotine Dependence; RCQ = Readiness to Change Questionnaire.

**Table S2** Summary statistics on outcomes by Condition and Time in ITT sample (*N* = 77)

| Outcomes/Time | ApBM+  (*n* = 29) | Standard-ApBM  (*n* = 24) | Sham-ApBM  (*n* = 24) | *F*(2, 74) | *p-value* |
| --- | --- | --- | --- | --- | --- |
| ApB for smoking, *M* (*SD*) |  |  |  |  |  |
| Pre-test | 12.32 (71.38) | 2.54 (42.26) | -0.56 (63.61) | 0.33 | 0.723 |
| Post-test | -26.69 (70.91) | -18.93 (39.24) | -8.77 (63.79) | - | - |
| 1-month follow-up | -5.41 (80.06) | -5.85 (48.13) | 1.85 (54.32) | - | - |
| ApB for alternative activities, *M* (*SD*) | | | | | |
| Pre-test | -2.84 (55.12) | 8.32 (47.84) | -8.97 (52.44) | 0.68 | 0.509 |
| Post-test | -19.10 (68.17) | -10.21 (52.79) | 9.12 (61.78) | - | - |
| 1-month follow-up | 3.85 (40.27) | -4.96 (37.34) | 7.23 (49.79) | - | - |
| ApB for neutral activities, *M* (*SD*) | | | | | |
| Pre-test | -2.55 (57.64) | -3.58 (56.04) | 0.30 (43.87) | 0.04 | 0.966 |
| Post-test | -16.28 (51.79) | -17.99 (38.41) | 3.98 (79.40) | - | - |
| 1-month follow-up | 7.80 (36.45) | -3.28 (41.90) | 5.10 (54.31) | - | - |
| QSU, *M* (*SD*) |  |  |  |  |  |
| Pre-test | 25.52 (7.97) | 30.96 (11.89) | 26.54 (8.51) | 2.34 | 0.104 |
| Post-test | 16.36 (8.01) | 20.30 (10.81) | 17.22 (8.12) | - | - |
| 1-month follow-up | 14.11 (6.24) | 18.44 (11.01) | 18.10 (7.89) | - | - |
| DCC, *M* (*SD*) |  |  |  |  |  |
| Pre-test | 13.76 (7.23) | 14.39 (7.02) | 13.55 (4.92) | 0.11 | 0.897 |
| Post-test | 4.34 (6.80) | 3.93 (6.51) | 3.68 (5.70) | - | - |
| 1-month follow-up | 5.06 (5.31) | 4.39 (6.22) | 5.50 (6.31) | - | - |
| CO levels, *M* (*SD*) |  |  |  |  |  |
| Pre-test | 14.45 (8.55) | 14.67 (6.83) | 14.25 (7.37) | 0.02 | 0.982 |
| Post-test | 6.22 (7.53) | 7.20 (7.62) | 6.10 (5.87) | - | - |
| 1-month follow-up | 5.55 (4.87) | 5.85 (5.60) | 7.09 (6.72) | - | - |
| 7D-PPA, *n*_1_*/n*_2_^a^ *(%)* |  |  |  |  |  |
| Pre-test | 0 (0.00) | 0 (0.00) | 0 (0.00) | - | - |
| Post-test | 8/29 (27.6) | 6/24 (25.0) | 6/24 (25.0) | - | - |
| 1-month follow-up | 5/29 (17.2) | 5/24 (20.8) | 5/24 (20.8) | - | - |

*Note.* ITT = intention-to-treat analyses. ApBM = Approach Bias Modification; ApB = approach biases; QSU =

Questionnaire on Smoking Urge; DCC = daily cigarette consumption; CO levels = breath carbon monoxide

level; 7D-PPA = 7-day point prevalence abstinence. ^a^the number of participants coded as 1 (i.e., quit) at a test

time-point divided by the total number of participants who reported their smoking status at the same test

time-point.

**Results**

**Training effects at post-test**

***Primary outcomes: Approach biases***

Results showed a main effect of Time in reducing ApB for smoking pictures from pre-test to post-test (*F*(1,66.65) = 7.21, *p* = 0.009), but no interaction with Condition (*F*(2,71.41) = 1.13, *p* = 0.328; see Table S2). Regarding changes of ApB for alternative-activity pictures and for neutral-activity pictures from pre-test to post-test, there was neither a significant effect of Time (alternative-activity pictures: *F*(1,62.14) = 0.41, *p* = 0.523; neutral-activity pictures: *F*(1,66.98) = 0.87, *p* = 0.355) nor any significant interactions with Condition (alternative-activity pictures: *F*(2,70.45) = 1.79, *p* = 0.174; neutral-activity pictures: *F*(2,72.42) = 0.43, *p* = 0.649; see Table S2).

Moderation analyses revealed an interaction effect of Condition and severity of smoking at a statistical trend level regarding ApB for smoking pictures (*F*(2,70) = 3.23, *p* = 0.054). This effect was not found for ApB for alternative-activity or neutral-activity pictures (*p*s ≥ 0.193). Specifically, from pre-test to post-test, ApBM+ was more effective in reducing ApB for smoking than sham-ApBM for relatively heavy smokers (*b* = -43.74, *95% CI* = [-80.00, -7.48], *p* = 0.018), but this difference was not found between ApBM+ and standard-ApBM (*b* = -11.48, *95% CI* = [-44.68, 21.72], *p* = 0.497), or between standard-ApBM and sham-ApBM (*b* = -32.26, *95% CI* = [-70.59, 6.08], *p* = 0.100; see Fig. S3). When considering the simple slopes (see Fig. S3), it is noteworthy to mention, first, that after receiving sham-ApBM, the ApB for smoking increased in heavy smokers, but it decreased in light smokers (*b* = 30.12, *95% CI* = [0.77, 59.46], *p* = 0.044); second, after receiving ApBM+, the ApB for smoking reduced more in heavy smokers than in light smokers (*b* = -13.63, *95% CI* = [-34.91, 7.66], *p* = 0.210), but this latter effect was not significant.


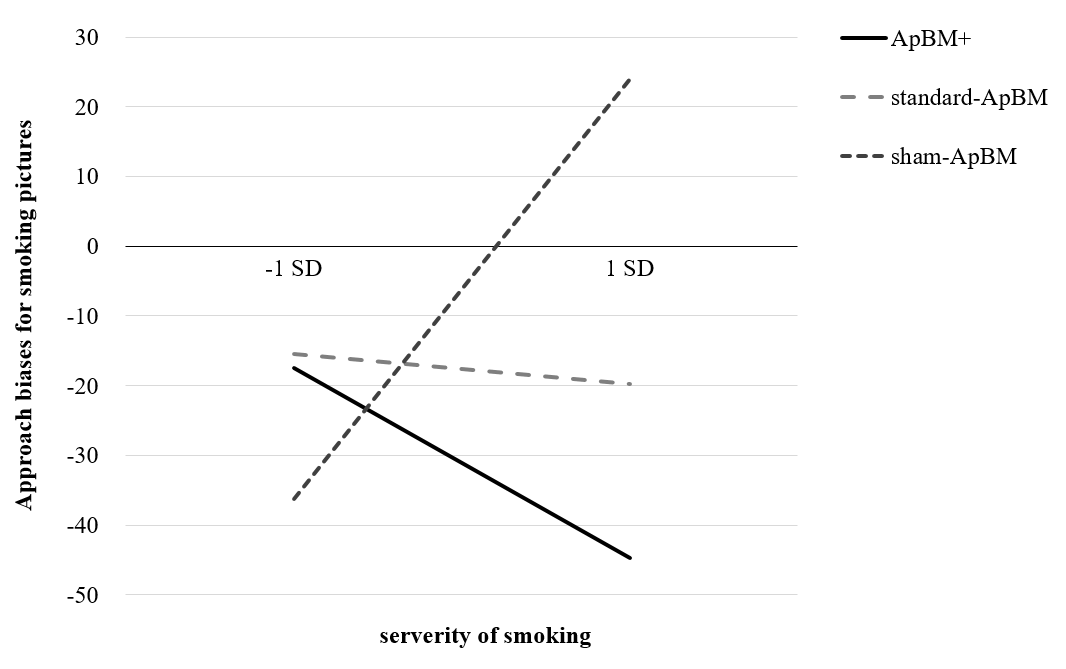


**Fig. S3** The interaction between Condition and severity of smoking (-1 SD vs. +1 SD) in predicting approach biases for smoking pictures (higher index indicates stronger biases) at post-test.

*Note.* ApBM = Approach Bias Modification. The results were based on ITT analyses.

***Secondary outcomes: Smoking-related Behaviors***

Results showed a main effect of Time in reducing craving (*F*(1,63.51) = 50.40, *p* < 0.001), daily cigarette consumption (*F*(1,65.48) = 141.54, *p* < 0.001), and CO levels (*F*(1,59.78) = 71.37, *p* < 0.001) from pre-test to post-test, in the absence of interactions with Condition (*p*s ≥ 0.876; see Table S2). The 7-day point prevalence abstinence rates at post-test did not differ across training conditions (*F*(2, 9730.56) = 0.07, *p* = 0.930; see Table S2). Additionally, moderation analyses showed no significant interaction effects of Condition and severity of smoking on all smoking-related behavior outcomes at post-test (*p*s ≥ 0.265).

**Training effects at 1-month follow-up**

The results demonstrated no interactions between Condition and Time on ApB, craving, daily cigarette consumption, CO levels at 1-month follow-up, and 7-day point prevalence abstinence rates at 1-month follow-up did not differ across training conditions. There was only a main effect of Time in reducing craving, daily cigarette consumption, and CO levels from pre-test to 1-month follow-up (see Table S2). Additionally, moderation analyses showed no significant interaction effects of Condition and severity of smoking on all outcomes at 1-month follow-up.

**Electronic Supplementary Material 7: Additional exploratory analysis: Whether severity of smoking moderated the effects of Mental Imagery Procedure (MIP) on craving?**

Based on our exploratory moderation analyses, there was an indication of a difference in the change of smoking-approach biases between sham-ApBM and ApBM+ for relatively heavy smokers at post-test. This was primarily driven by a significant increase in smoking-approach biases in the sham-ApBM and a trend decrease in smoking-approach biases in the ApBM+. We proposed that one possible explanation for this divergent training effect in relatively heavy smokers could be that compared to light smokers, heavy smokers may have higher levels of craving as a training context regardless of their training condition. Thus, the higher state of craving in relatively heavy smokers may be beneficial when receiving ApBM+ but detrimental when receiving sham-ApBM. In order to investigate this possibility, we examined whether the severity of smoking moderated the MIP effects in changing craving***.*** Given our results, showing that MIP led to increased craving in ApBM+ and decreased craving in both standard-ApBM and sham-ApBM, we further expected that for heavy smokers compared to light smokers in the ApBM+ condition, MIP would lead to greater increases in craving, while in the sham-ApBM condition leading to lesser decreases. As a result, in both ApBM+ and sham-ApBM conditions, heavy smokers would have higher craving levels than light smokers after the MIP (i.e., before conducting ApBM training).

To test whether severity of smoking moderated MIP effects, we conducted a one-way repeated ANOVA with covariates for each training condition on the average craving scores across training sessions, separately. Specifically, in each model, we entered Time as a within-subject factor (before MIP vs. after MIP), severity of smoking as a covariate, and their interaction term. We especially focused on the interaction effect of Time and severity of smoking on craving changes. The results showed that in the ApBM+ condition, there was a significant Time and severity of smoking interaction effect (*F*(1,23) = 8.23, *p* = 0.009, η_p_^2^ = 0.263); while this interaction effect was not found in the standard-ApBM condition (*F*(1,17) = 0.08, *p* = 0.784, η_p_^2^ = 0.005) or in the sham-ApBM condition (*F*(1,20) = 1.48, *p* = 0.238, η_p_^2^ = 0.069). Therefore, severity of smoking moderated the MIP effects only in the ApBM+ condition. However, the results were not in line with our expectations. Specifically, in the ApBM+ condition, craving increased from before to after MIP for light smokers, but this change was not observed for heavy smokers (see Fig. S4). The later lack of effect was most likely driven by the fact that heavy smokers in the ApBM+ had already shown relatively high levels of craving before conducting the MIP. Therefore, the MIP used in this study may not have been strong enough to produce further cue-evoked craving, especially in the case of heavy smokers (see relevant discussion points within the main manuscript).

Despite the moderation effects of severity of smoking on MIP effects not being in line with our expectations, we did notice that, descriptively, heavy smokers indeed showed higher levels of craving than light smokers in both ApBM+ and sham-ApBM conditions after the MIP (i.e., before conducting the ApBM training; see Fig. S4). Thus, this might still support our idea that higher levels of craving as a training context led relatively heavy smokers to improve from ApBM+ but worsen from sham-ApBM.


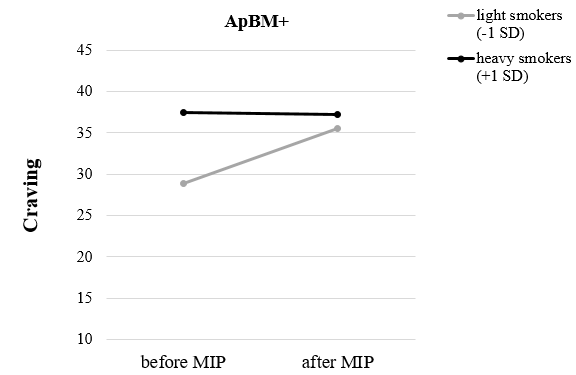


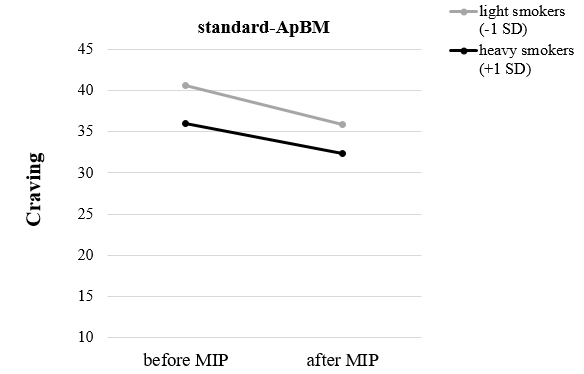


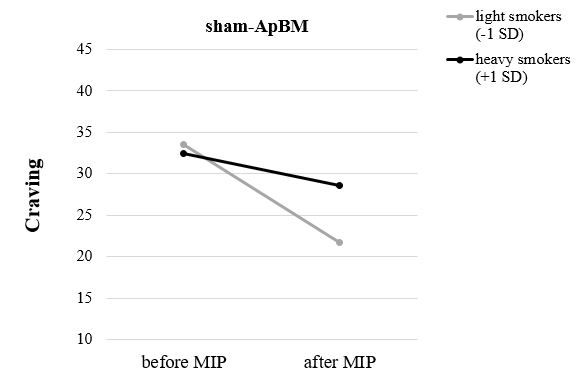


**Fig. S4** The interaction between Time (before MIP vs. after MIP) and severity of smoking (-1 SD vs. +1 SD) in predicting craving (higher index indicates stronger levels) in each condition.

*Note.* ApBM = Approach Bias Modification. MIP = Mental Imagery Procedure.

**References**

1. Parsons S, Kruijt AW, Fox E. Psychological Science needs a standard practice of reporting the reliability of cognitive behavioural measurements. 2018. https://doi.org/10.31234/OSF.IO/6KA9Z

2. Parsons S. splithalf: robust estimates of split half reliability (Version 2). figshare. 2017. https://doi.org/doi:10.6084/m9.figshare.5559175.v2

3. Wittekind CE, Lüdecke D, Cludius B. Web-based Approach Bias Modification in smokers: A randomized-controlled study. *Behav Res Ther*. 2019;116:52–60.

4. Wittekind CE, Reibert E, Takano K, Ehring T, Pogarell O, Rüther T. Approach-avoidance modification as an add-on in smoking cessation: A randomized controlled study. *Behav Res Ther*. 2019;114:35–43.

5. Kong G, Larsen H, Cavallo DA, Becker D, Cousijn J, Salemink E, et al. Re-training automatic action tendencies to approach cigarettes among adolescent smokers: A pilot study. *Am J Drug Alcohol Abuse.* 2015;41:425–32.

6. Ataya AF, Adams S, Mullings E, Cooper RM, Attwood AS, Munafò MR. Internal reliability of measures of substance-related cognitive bias. *Drug Alcohol Depend.* 2012;121:148–51.

7. Groefsema M, Engels R, Luijten M. The role of social stimuli content in neuroimaging studies investigating alcohol cue-reactivity. *Addict Behav.* 2016;58:123–8.

8. Morris TP, White IR, Royston P. Tuning multiple imputation by predictive

mean matching and local residual draws. *BMC Med Res Methodol*. 2014;14:75.

9. Grund S, Lüdtke O, Robitzsch A. Multiple imputation of multilevel missing data: An

introduction to the R package pan. *Sage Open*. 2016;6.

10. van Ginkel JR, Kroonenberg PM. Analysis of variance of multiply imputed data.

*Multivariate Behav Res*. 2014;49:78-91.

11. van Ginkel, JR. SPSS syntax for applying rules for combining multivariate estimates in

multiple imputation. *Leiden University*. 2014.

12. van Ginkel JR, Linting M, Rippe RC, van der Voort A. Rebutting existing

misconceptions about multiple imputation as a method for handling missing data. *J Pers*

*Assess*. 2020;102:297-308.
